# Improving type 2 diabetes detection among at-risk individuals – comparing the effectiveness of active opportunistic screening using spot capillary-HbA1c testing and venous HbA1c testing: a cluster randomized controlled trial

**DOI:** 10.1186/s12916-025-04007-z

**Published:** 2025-03-31

**Authors:** Linda Chan, Esther Yee Tak Yu, Eric Yuk Fai Wan, Samuel Yeung Shan Wong, David Vai Kiong Chao, Welchie Wai Kit Ko, Catherine Xiao Rui Chen, Paul Po Ling Chan, Emma Victoria Marianne Bilney, Eng Sing Lee, Wei Leik Ng, Cindy Lo Kuen Lam

**Affiliations:** 1 Department of Family Medicine and Primary Care, Ap Lei Chau Clinic, The University of Hong Kong, 3/F, 161 Main Street, Ap Lei Chau, Hong Kong SAR, China; 2 The Bau Institute of Medical and Health Sciences Education, The University of Hong Kong, Hong Kong SAR, China; 3 Department of Family Medicine and Primary Care, The University of Hong Kong-Shenzhen Hospital, Shenzhen, Guangdong China; 4 Health Bureau, Primary Healthcare Commission, Hong Kong SAR, China; 5 Centre for Safe Medication Practice and Research, Department of Pharmacology and Pharmacy, The University of Hong Kong, Hong Kong SAR, China; 6 The Jockey Club School of Public Health and Primary Care, The Chinese University of Hong Kong, Hong Kong SAR, China; 7 Department of Family Medicine and Primary Health Care, United Christian Hospital, Kowloon East Cluster, Hong Kong Hospital Authority, Hong Kong SAR, China; 8 Lee Kong Chian School of Medicine, Nanyang Technology University, Singapore, Singapore; 9 Department of Primary Care Medicine, Faculty of Medicine, Universiti Malaya, Kuala Lumpur, Malaysia; 10 The Institute of Cardiovascular Science and Medicine, The University of Hong Kong, Hong Kong SAR, China; 11 Advanced Data Analytics for Medical Science Limited, Hong Kong SAR, China; 12 Hong Kong Hospital Authority, Hong Kong SAR, China

**Keywords:** Type 2 diabetes, Screening, Point-of-care capillary HbA1c, POC-cHbA1c, Primary care

## Abstract

**Background:**

Delayed diagnosis and treatment of type 2 diabetes increases diabetes-related complications, making the high prevalence of undiagnosed type 2 diabetes in Hong Kong an important concern. Point-of-care capillary HbA1c (POC-cHbA1c) testing holds promise as a comparably accurate, convenient, and timely alternative to venous HbA1c (vHbA1c) for type 2 diabetes screening, yet randomized trials are lacking. This study compared the effectiveness of a 2-step active opportunistic screening strategy using POC-cHbA1c versus usual practice employing vHbA1c and multiple clinic visits in detecting type 2 diabetes among at-risk primary care patients. The primary outcomes were to identify the difference in the proportion of type 2 diabetes detected between intervention (POC-cHbA1c) and control (vHbA1c) groups and the uptake rate of POC-cHbA1c versus vHbA1c testing among consenting participants.

**Methods:**

A cluster randomized controlled trial was conducted in 8 General Out-Patient Clinics between June 2022 and January 2024 using 2-step active opportunistic screening.

In step 1, risk factor count, 852 at-risk patients were identified through consecutive sampling during their primary care consultation by specific inclusion and exclusion criteria. In step 2, these at-risk patients then underwent POC-cHbA1c (intervention) or vHbA1c (control) testing. If preliminary HbA1c was ≥ 5.6%, a confirmatory oral glucose tolerance test was offered. Randomization occurred at the clinic level using a random allocation sequence generated by statistical software. Multilevel logistic regression analyses were employed to evaluate the effect of the intervention on the uptake rate, adjusting for patient characteristics and clinic clustering.

**Results:**

POC-cHbA1c had a higher uptake rate than vHbA1c (76.0% vs 37.5%; OR = 7.06, 95% CI [2.47–20.18], *p* < 0.001). A greater proportion of type 2 diabetes (4.2% vs 1.4%; *p* = 0.016) and pre-diabetes (11.8% vs 6.9%; *p* = 0.015) were detected using POC-cHbA1c versus vHbA1c. POC-cHbA1c was more likely to detect type 2 diabetes/pre-diabetes combined (OR = 1.99, 95% CI [1.01–3.95], *p* = 0.048). The number-needed-to-screen to detect one additional type 2 diabetes patient with POC-cHbA1c was 61 versus vHbA1c.

**Conclusions:**

POC-cHbA1c testing was associated with a higher uptake rate and detection of type 2 diabetes versus vHbA1c, underscoring its potential as an effective type 2 diabetes screening strategy in primary care.

**Trial registration:**

NCT06382363 (retrospectively registered: 2024–04-19).

**Supplementary Information:**

The online version contains supplementary material available at 10.1186/s12916-025-04007-z.

## Background

Globally, type 2 diabetes affects over 500 million people, more than half of whom live in Asia [1]. In Hong Kong (HK), the prevalence of type 2 diabetes is escalating, affecting 8.5% of the general [2], and 14.6% of the at-risk [3] populations. Due to its asymptomatic nature early on, type 2 diabetes can remain unrecognized for prolonged periods [4], potentially masking its true prevalence. As of 2020, approximately 36.6% of people with type 2 diabetes in HK were undiagnosed [2], twice the rate of the United Kingdom [5]. Delayed diagnosis increases the risk of diabetes-related macro- and micro-vascular complications and mortality [6]. Effective type 2 diabetes screening is crucial for early diagnosis, prevention of complications [7], and to reduce the burden on patients and healthcare systems. However, type 2 diabetes screening in HK is primarily provided by the private sector at the patient’s own cost, with public primary care clinics (i.e. General Out-Patient Clinics; GOPCs) offering annual low-cost screenings only to those with hypertension, a type 2 diabetes risk factor. This approach results in delayed diagnosis [8], predominantly impacting at-risk populations who cannot afford the cost and time for private body checks and thus, are unlikely to receive screening appropriate to their risk [9]. Recognizing this, a Chronic Disease Co-Care (CDCC) Pilot Scheme has recently been implemented [10]. This facilitates subsidized screening for and management of specific chronic conditions like type 2 diabetes within the private healthcare sector, in conjunction with the District Health Center or District Health Center Express. Yet, the CDCC pilot scheme still heavily depends on eligible individuals being willing to participate and having proactive help-seeking behaviors. Therefore, a more accessible public screening strategy is urgently needed to reduce the prevalence of undiagnosed type 2 diabetes with its deleterious consequences.

The choice of screening test could be implicated in the high local prevalence of undiagnosed type 2 diabetes. The World Health Organization (WHO) [11] and American Diabetes Association (ADA) [12] recommend three screening and diagnostic tests: fasting plasma glucose (FPG), oral glucose tolerance test (OGTT) and glycated hemoglobin (HbA1c). While OGTT remains the gold standard [11–13], FPG is the most common screening test in HK due to its low cost [14, 15] and OGTT is reserved as the confirmatory diagnostic test [12–16]. However, FPG has limited sensitivity (50%) in detecting diabetes at the recommended cut-off (≥ 7.0 mmol/L) [15–17]. Alternatively, venous HbA1c (vHbA1c) is a relatively stable diagnostic measure that does not require fasting and yields comparable results in predicting diabetic retinopathy development versus FPG [11–16]. However, due to logistical challenges in HK public primary care clinics, vHbA1c testing is seldom performed in the same clinical visit.

Recently available National Glycohemoglobin Standardization Program (NGSP)-certified point-of-care (POC) HbA1c assays using capillary blood (cHbA1c) present a promising solution [12] by providing rapid on-site results without venipuncture [17]. Globally, POC-cHbA1c has generated interest as a potential suitable screening and diagnostic test for type 2 diabetes [17]. Community-based POC-cHbA1c testing has been demonstrated to be feasible and acceptable to patients, with comparable accuracy and precision to conventional laboratory measurements [18–23]. However, POC-cHbA1c testing is currently limited in primary care clinics to monitoring glycemic control in diabetic patients.

This study proposes a 2-step active opportunistic screening strategy in primary care combining risk factor assessment with HbA1c testing to increase the uptake of confirmatory OGTT among at-risk individuals. Multi-step opportunistic screening tailored to specific subpopulations and conducted as part of routine primary care practice has been reported to increase OGTT uptake and the type 2 diabetes detection rate [12, 24–26]. POC-cHbA1c testing is hypothesized to be superior to vHbA1c due to its convenience, and generation of immediate results to support diagnosis among at-risk populations. Despite its potential, there have been few randomized controlled trials globally, and none conducted in HK. Therefore, this study aims to evaluate the effectiveness of a 2-step active opportunistic screening approach using POC-cHbA1c compared to vHbA1c testing among at-risk individuals in primary care. The findings will provide insights into whether this strategy enhances type 2 diabetes screening uptake and detection among at-risk HK public primary care patients.

## Methods

### Study design and participants

A cluster randomized controlled trial (cRCT) was conducted to evaluate the effectiveness of a 2-step active opportunistic screening strategy using POC-cHbA1c testing compared to vHbA1c, in enhancing the detection of type 2 diabetes among public primary care patients with type 2 diabetes risk factors. We designed the trial to determine differences between POC-cHbA1c (intervention) and vHbA1c (control) testing in terms of: i) HbA1c uptake rate; ii) proportion of type 2 diabetes detected; and iii) uptake rate of confirmatory OGTT. We hypothesized that POC-cHbA1c testing would have higher uptake plus OGTT testing rates and would identify a greater proportion of type 2 diabetes patients compared to vHbA1c among the at-risk population. The trial involved 4 HK Hospital Authority clusters, with 2 GOPCs selected within each cluster resulting in a total of 8 participating GOPCs (Additional file 1: Table S1). Randomization occurred at the clinic level to minimize contamination between participants within the same clinic and this randomization was conducted using a random allocation sequence generated by statistical software. Each clinic was randomly assigned a code of either 0 (control) or 1 (intervention) by the study statistician (E.Y.F.W) using a matched design. The sequence generation and randomization were conducted independently by the study statistician who remained blinded to the identity of the GOPCs throughout the process. This ensured an unbiased allocation, resulting in 4 intervention and 4 control clinics, one from each cluster. All recruited subjects from the same GOPC were assigned to the same group, ensuring a cluster-based approach.

Our participants were public primary care patients who were at-risk for type 2 diabetes according to the screening criteria of the WHO/International Diabetes Federation (IDF) [27] and Hong Kong Reference Framework for Diabetes Care for Adults in Primary Care Settings [28]. At-risk participants without any of the exclusion criteria were eligible for our study. Eligible participants were identified during primary care consultations through consecutive sampling using a standardized eligibility screening form listing inclusion and exclusion criteria (Additional file 1: Appendix 1). Eligible patients were informed about their type 2 diabetes risk and offered free testing (i.e. either POC-cHbA1c at intervention or vHbA1c at control clinics). Statistical considerations for sample size calculation and the analysis plan were set before the data were collected.

### Sample size

The sample size was estimated based on the difference in the prevalence of type 2 diabetes to be detected between the intervention and control groups. For the control group, it was estimated that 30% of participants would agree to undergo a free venous HbA1c (vHbA1c) test if advised by their doctor. This assumption was derived from data in the Hong Kong Population Health Survey 2014–15 conducted by the Department of Health of the HK Special Administrative Region government, which found that only 57.7% of respondents had ever undergone glucose testing for type 2 diabetes, and only 37.6% had regular body checks [9]. Additionally, findings from the 2009 Thematic Household Survey Report indicated that the proportion was expected to increase as the screening test was offered for free or suggested by healthcare professionals [29]. Given that the study targeted individuals who were unable to afford regular body checks, 30% uptake of the vHbA1c test was considered a conservative estimate for the control group. For the intervention group, the uptake rate was assumed to be much higher at 70%. This estimate reflects the additional benefits of the point-of-care capillary HbA1c (POC-cHbA1c) test compared to the vHbA1c test used in the control group. Specifically, the POC-cHbA1c test eliminates barriers such as the fear of venipuncture and the time commitment required for conventional blood tests. The immediate availability of test results was also expected to motivate individuals who were curious about their health to participate. Therefore, a 70% uptake rate was regarded as a reasonable assumption for participants offered the POC-cHbA1c test on the day of recruitment.

The proportion of participants expected to screen positive (i.e., HbA1c ≥ 5.6%) was estimated to be the same for both groups because the participants in both arms were drawn from similar populations with comparable risk factors. Previous studies showed a range of prevalence for pre-diabetes depending on the test and cutoff used, with 14.0% of the general population demonstrating fasting plasma glucose (FPG) concentrations between 5.6–7.0 mmol/L or HbA1c levels between 6.0–6.5% [9]. Additionally, among primary care patients with hypertension and impaired fasting glucose (IFG), 85.5% were found to have HbA1c ≥ 5.6% [30]. Since this study’s target population consisted of older adults with higher body mass index (BMI) than the general population but did not universally include individuals with hypertension or IFG, a conservative estimate of 45% screening positive was applied for both groups.

Among participants screening positive, it was estimated that 80% would subsequently agree to take an oral glucose tolerance test (OGTT) [25, 30]. This estimate aligns with participation rates reported in prior research conducted in primary care settings. Additionally, based on the results of an earlier study, 22.7% of individuals with HbA1c ≥ 5.6% were expected to be diagnosed with type 2 diabetes following the OGTT [30]. Combining these proportions, it was estimated that the diabetes detection rate in the control group would be approximately 2%, while the intervention group would have a higher detection rate of 5%. Finally, considering the cluster effect of the cRCT, the common intra-cluster correlation coefficient in primary care research ranged from 0 to 0.021 (median 0.005) [31]. Assuming an intra-cluster correlation coefficient of 0.005 in this study, a total of 776 participants from the 8 clinics (97 per clinic) would be required to detect the difference between groups based on a power of 80% and significance level of 0.05 [32]. The power calculations for the two primary outcomes, namely the HbA1c uptake rate and the proportion of type 2 diabetes detected, were 100% and 70.3% respectively [33].

### Procedures

This cRCT consisted of a 2-step active opportunistic screening strategy. Additional file 1: Fig. 1 illustrates our study flow. During step 1, all public primary care patients underwent active opportunistic risk factor screening. Eligible participants signed written consent for inclusion. Sociodemographic, lifestyle, medical and medication history, plus health service utilization information were collected on a standardized questionnaire by a research assistant, along with blood pressure, body height, body weight, BMI, hip girth and waist circumferences. Subsequently, participants underwent step 2 i.e., either POC-cHbA1c at intervention or vHbA1c at control clinics. Additional file 1: Appendix 2 details the protocol for all tests including staff training, quality assurance measures, stepwise procedures, and standardization processes.

Eligible participants in the intervention clinics were offered free POC-cHbA1c testing. This was conducted on-site during the same recruitment visit using the Cobas b 101 diagnostic test system (Roche Diagnostics, Switzerland), to measure the HbA1c level in a capillary blood sample by photometric transmission. Testing was carried out by nurses and researchers who had been trained by the Cobas b 101 machine supplier in the use of the POC-cHbA1c machine and internal quality assurance processes. This training encompassed proper device operation, sample collection techniques, adherence to quality control protocols, and troubleshooting procedures. Internal and external quality assurance measures were also implemented.

For internal quality assurance, this involved an optical test and a quality control (QC) test which were conducted plus documented by the attending trained nurse or researcher at the start of each session when the Cobas b 101 machine was used for POC-cHbA1c testing. The optical test verified the optical functions of the machine and only when this was passed, would the trained nurse or researcher proceed to the QC test. The QC test ensured that the operation technique plus results obtained from the machine were accurate. Testing of patients was only initiated if both the optical and QC tests yielded results within acceptable ranges.

External quality assurance was ensured through monthly proficiency tests. The proficiency test verified if the POC-cHbA1c test system, operation technique, reagents, and testing performance were comparable with external operators. A supporting laboratory provided blinded quality control samples to the trained nurse or researcher conducting the POC-cHbA1c test and the results from different nurses or researchers were analyzed against each other. Monthly reports detailing these comparisons of peer performance were reviewed alongside internal quality assurance records to ensure ongoing compliance with rigorous testing standards, with additional training mandated for those with unsatisfactory performance.

To ensure accuracy and reliability of the POC-cHbA1c test results, measurements were performed using the NGSP-certified Cobas b 101 machine, and the internal plus external quality assurance tests described above were conducted regularly according to protocols detailed in Additional file 1: Appendix 2. For the stepwise POC-cHbA1c testing procedure, participants were instructed to cleanse their hands with non-alcoholic soap before drying thoroughly. A capillary blood sample was obtained by dermal puncture of a fingertip using a disposable lancet and absorbed within the designated section of the test disc, which was subsequently placed into the autoanalyzer. Results displayed in both percentage and mmol/L were available within 10 min. Participants with HbA1c levels ≥ 5.6% were immediately informed of their type 2 diabetes risk and offered a confirmatory OGTT, scheduled to occur within 2–4 weeks at the same clinic.

Eligible participants recruited in control clinics were offered free vHbA1c testing. Consistent with current practice in GOPCs, the vHbA1c test was scheduled for a separate clinic visit within 1–2 weeks of recruitment. Trained nurses collected non-fasting venous blood samples, which were then sent to a laboratory accredited by the HK Accreditation Service and/or an international college of pathologists (e.g., The College of American Pathologists or The Royal College of Pathologists of Australasia). High-performance liquid chromatography was used for vHbA1c concentration measurement. Results were available within one week and participants were informed of their type 2 diabetes risk by phone. Those with HbA1c levels ≥ 5.6% were scheduled for a confirmatory OGTT within 2–4 weeks at the same clinic.

HbA1c level ≥ 5.6% was used to determine type 2 diabetes risk as it has the highest sensitivity (96.1%) and negative predictive value (94.5%) in detecting type 2 diabetes among the local population [30]. Participants from both intervention and control clinics with high-risk HbA1c levels ≥ 5.6% were offered a free confirmatory 75-g OGTT, before which they were advised to maintain a normal diet for 3 days then strictly fast for 8 h prior to blood sampling. During the OGTT, they first underwent fasting venous blood sampling, followed by the consumption of a 300 ml solution containing 75-g of glucose within 5 min. Participants were instructed to remain seated and refrain from eating or smoking until the second venous blood sampling after 2 h. The paired blood samples were dispatched to an accredited laboratory for analysis using the Hexokinase method. The diagnosis of type 2 diabetes (epidemiological) was defined by the OGTT test result, according to ADA [12] and WHO/IDF [27] criteria: FPG ≥ 7.0 mmol/L and/or 2-h post-challenge plasma glucose concentration ≥ 11.1 mmol/L. Physicians of confirmed type 2 diabetes participants were informed of their diagnosis for further management.

### Outcomes

The primary outcomes were: i) the difference in the proportion of type 2 diabetes detected between the intervention (POC-cHbA1c) and control (vHbA1c) clinics; and ii) the uptake rate of POC-cHbA1c and vHbA1c testing among consenting participants. Our prespecified secondary outcomes were: i) the number-needed-to-screen (NNS) using POC-cHbA1c testing to detect one more type 2 diabetes case compared to vHbA1c; ii) the proportion of participants with high-risk HbA1c ≥ 5.6% among the study’s at-risk group; and iii) the difference in uptake rates of confirmatory OGTT between the intervention and control clinics. Additional outcomes included the difference in proportion of pre-diabetes (i.e., impaired fasting glucose [IFG]/impaired glucose tolerance [IGT]) detected between the intervention and control clinics, and the difference in the proportion of type 2 diabetes plus pre-diabetes combined detected between the intervention and control clinics.

### Statistical analysis

Descriptive statistics summarized patients’ characteristics in the intervention and control clinics. Between-group differences in characteristics were assessed using independent t-tests for continuous variables or Chi-square tests for categorical variables, respectively. The uptake rates of POC-cHbA1c and vHbA1c testing were reported. The difference in the proportion of type 2 diabetes detected between groups was compared using a Chi-squared test. The NNS to identify one additional patient with type 2 diabetes using POC-cHbA1c compared to vHbA1c was calculated (i.e., NNS = 1/Absolute Risk Reduction [ARR], where ARR was calculated as the difference between the incident rate between the intervention and control groups).

Outcome variables (i.e., HbA1c uptake, overall detection, and OGTT uptake rates) were first examined to determine whether they could meet assumption requirements primarily pertaining to issues such as non-normal distribution, heteroscedasticity, or violation of the independence assumption. The degree to which clinic-level factors influenced outcomes was then assessed by examining variations across clinics that could not be attributed to random chance alone. Variables that did not meet the assumptions or had no evidence of substantial clustering between clinics were excluded. Mixed-effects logistic regression analyses were conducted to evaluate the effect of the intervention on the HbA1c uptake rate, overall detection rate (type 2 diabetes/pre-diabetes), and OGTT uptake rate, with the clinic treated as the random effect and adjusting for the patients’ characteristics. HbA1c uptake, overall detection, and OGTT uptake were generated as binary variables i.e., HbA1c uptake was indicated by 1 or 0 (no uptake); detection of either type 2 diabetes or pre-diabetes was indicated by 1 or 0 (not detected); and OGTT uptake was indicated by 1 or 0 (no uptake). For all analyses, we adjusted for participant characteristics that may influence type 2 diabetes screening and diagnostic uptake or detection. These included: *gender, age, drinking status, smoking status, BMI, number of type 2 diabetes risk factors,* plus the following specific type 2 diabetes risk factors: *a first-degree relative with diabetes*, *a history of gestational diabetes*, *hypertension*, *IFG*, *IGT*, *hyperlipidemia*, and *obesity*. Due to multicollinearity of the variables *drinking status*, *smoking status*, *BMI,* and *number of type 2 diabetes risk factors*, these were removed from the analysis (Additional file 1: Table S2). With the exception of the *age* variable, all other variables were binary, categorized as 1 = positive and 0 = negative. The binary gender variable was categorized as 1 = Male and 2 = Female. Adjusted mixed-effects logistic regression models for dichotomous outcomes were used for all three outcome variables (HbA1c uptake, overall detection, and OGTT uptake rates) to account for clustering between clinics. Estimates of the fixed effects (coefficients) and the random effects (variance components) were collected at the clinic level.

All statistical analyses were conducted using IBM Statistical Package for the Social Sciences for Windows, version 28 (IBM Corp., Armonk, N.Y., USA). Cases with missing data were removed from the analysis. Adjusted point estimates (odds ratios [OR]) were determined with associated 95% confidence intervals (95% CI) and *p*-value. Two-tailed significance tests were used, with a *p*-value < 0.05 considered to be statistically significant.

### Ethics

Ethical approval was granted by the Institutional Review Boards of the 4 participating Hospital Authority clusters (Additional file 1: Table S1). Our study was registered with ClinicalTrials.gov (NCT06382363) and The University of Hong Kong Clinical Trials registries (HKUCTR-3002). Data collection followed the CONSORT guidelines for reporting randomized controlled trials (Additional file 1: Appendix 3) [34].

## Results

**Fig. 1:**
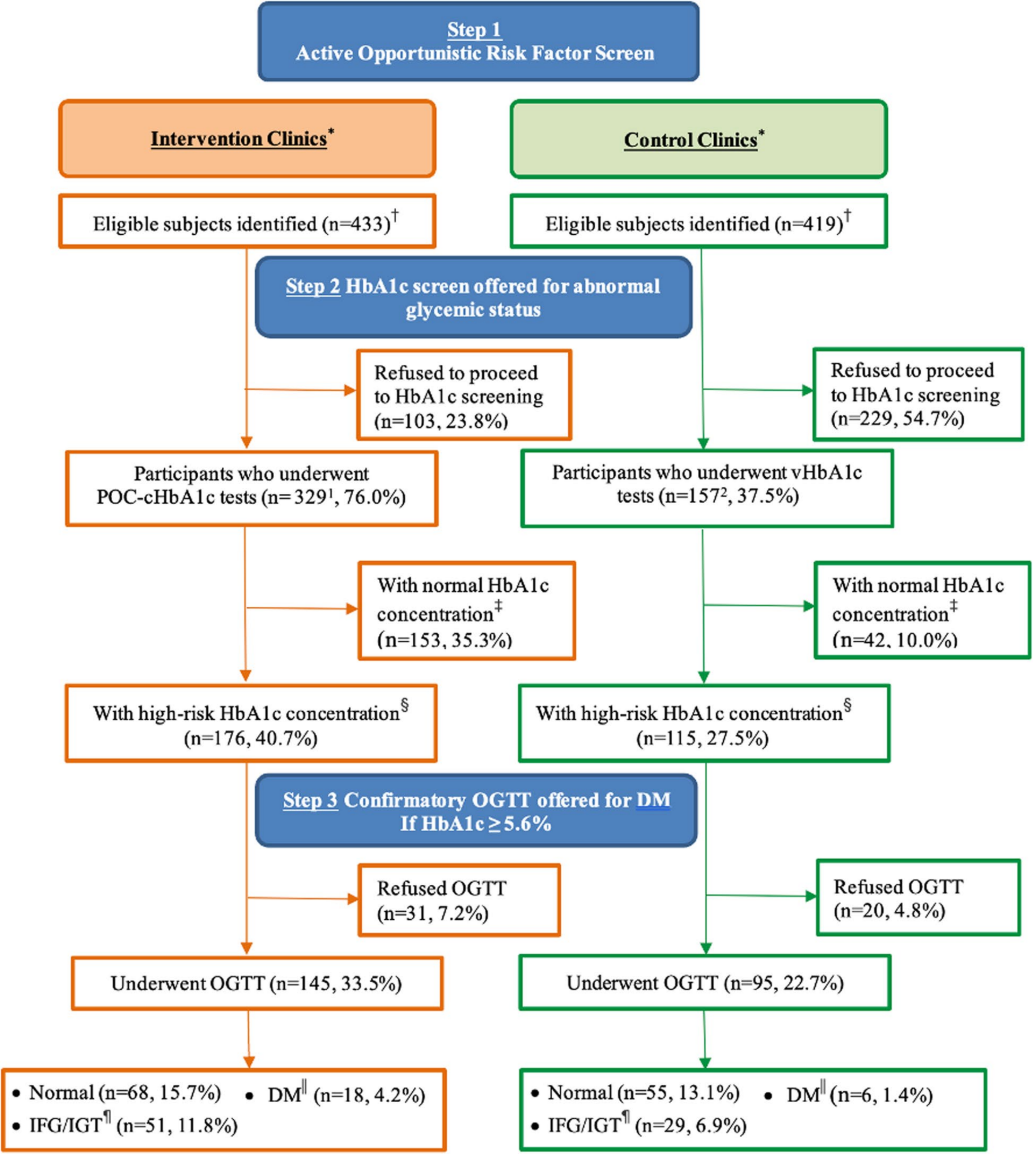
Study Flow Diagram. *Note.* HbA1c = Glycated hemoglobin; POC-cHbA1c = Point-of care capillary HbA1c; vHbA1c = venous HbA1c; OGTT = Oral glucose tolerance test; DM = Diabetes mellitus; IFG = Impaired fasting glucose; IGT = Impaired glucose tolerance. ^1^In total, 330 enrolled patients agreed to proceed to POC-cHbA1c screening, yet one patient refused to undergo POC-cHbA1c testing later. ^2^In total, 190 enrolled patients agreed to proceed to vHbA1c screening, yet 33 patients refused to undergo vHbA1c testing due to personal circumstances. ^*^Point-of care capillary HbA1c testing for intervention; venous HbA1c testing for control. ^†^Cases with missing data are removed from the analysis. ^‡^Normal HbA1c concentration refers to HbA1c < 5.6%. ^§^High-risk HbA1c concentration refers to HbA1c ≥ 5.6%. ^||^DM refers to fasting glucose ≥ 7.0 mmol/L and/or 2-h post-challenge plasma glucose concentration (2 h PG) ≥ 11.1 mmol/L according to the American Diabetes Association. ^¶^IFG (impaired fasting glucose) refers to fasting glucose between 5.6–6.9 mmol/L, and IGT (impaired glucose tolerance) refers to 2 h PG between 7.8–11.0 mmol/L according to the American Diabetes Association

Demographics of the 852 eligible participants included in the analyses are outlined in Additional file 1: Table S3. Within the intervention group, the majority engaged in active opportunistic screening, with more participants reporting a history of gestational diabetes, hypertension, and obesity. The distribution of individuals enrolled in each step of the active opportunistic screening process for both groups is summarized in Fig. 1. Characteristics of enrolled participants in each stage are detailed in Additional file 1: Tables S4-S6.

### Primary and secondary outcomes

In the intervention group, 329 of 433 eligible participants (76% uptake rate) underwent immediate POC-cHbA1c testing. Of these, 176 participants (40.7%) had high-risk HbA1c levels ≥ 5.6%, with 145 (33.5%) agreeing to undergo a confirmatory OGTT. Among them, 18 participants (4.2%) were confirmed to have type 2 diabetes and 51 (11.8%) were diagnosed with IFG/IGT. Whereas in the control group, only 157 of 419 eligible participants (37.5% uptake rate) underwent vHbA1c testing. Among them, 115 participants (27.5%) had HbA1c levels ≥ 5.6%, with 95 (22.7%) agreeing to undergo an OGTT. 6 participants (1.4%) were confirmed to have type 2 diabetes and 29 (6.9%) had IFG/IGT. Following the precedent set by existing screening literature [35], the NNS was used to quantify the effectiveness of the screening intervention, defined within our study as the number of participants needed to be screened to detect one additional case of type 2 diabetes using POC-cHbA1c testing compared to vHbA1c. As such, the NNS to detect one additional type 2 diabetes patient was 61 using POC-cHbA1c compared to vHbA1c testing.

Chi-squared analysis indicated a significant relationship between the intervention (POC-cHbA1c) and uptake of type 2 diabetes screening (*p* < 0.001), with intervention clinic participants more likely to undergo screening than those in the control clinics. Similarly, the intervention was significantly associated with type 2 diabetes detection (*p* = 0.016), indicating that a greater proportion of type 2 diabetes patients were detected using POC-cHbA1c compared to vHbA1c. Furthermore, a significant relationship existed between the diagnosis of pre-diabetes and the intervention (*p* = 0.015), with more cases detected using POC-cHbA1c. The overall detection rate (i.e., type 2 diabetes/pre-diabetes) was significantly higher in intervention than in control clinics (*p* < 0.001). Additionally, the relationship between POC-cHbA1c testing and the OGTT uptake rate was significant (*p* < 0.001).

### Mixed-effects logistic regression analyses

Mixed-effects logistic regression analyses, adjusting for patient characteristics and clinic clustering, were conducted following a statistically significant chi-squared result. Multicollinearity was absent for all three outcomes (Additional file 1: Table S2). Unconditional means models revealed substantial intra-class correlations (ICC) for the aforementioned outcomes as detailed in Additional file 1: Table S7.

#### HbA1c uptake rate

In the main adjusted model (Table 1), the baseline odds of uptake were significant (OR = 0.23, 95% CI [0.07–0.71], *p* = 0.011). POC-cHbA1c significantly increased the odds of uptake compared to vHbA1c (OR = 7.06, 95% CI [2.47–20.18], *p* < 0.001), holding other variables constant. Having a *first-degree relative with diabetes* (OR = 1.50, 95% CI [1.06–2.14], *p* = 0.024), *hyperlipidemia* (OR = 1.74, 95% CI [1.14–2.64], *p* = 0.010), or *obesity* (OR = 1.96, 95% CI [1.29–2.98], *p* = 0.002) significantly increased the likelihood of uptake. However, *hypertension* (OR = 0.45, 95% CI [0.31–0.65], *p* < 0.001) significantly decreased the odds of undergoing screening.

**Table 1:** Results of the two-level mixed-effects logistic regression model for the uptake rate of HbA1c testing

	***Uptake of Type 2 Diabetes Testing***	
	**OR [95% CI]**	***p***
***Fixed Effects***		
Intercept	0.23 [0.07–0.71]	0.011*
Intervention Condition (POC-cHbA1c)	7.06 [2.47–20.18]	0.001**
Gender (Female)	1.26 [0.92–1.74]	0.156
Age	1.01 [1.00–1.02]	0.100
First-Degree Relative with Diabetes (Yes)	1.50 [1.06–2.14]	0.024*
History of Gestational Diabetes (Yes)	1.78 [0.60–5.27]	0.300
Hypertension (Yes)	0.45 [0.31–0.65]	0.001**
Impaired Fasting Glucose (Yes)	0.99 [0.44–2.21]	0.970
Impaired Glucose Tolerance (Yes)	0.66 [0.07–6.53]	0.721
Hyperlipidemia (Yes)	1.74 [1.14–2.64]	0.010*
Obesity (Yes)	1.96 [1.29–2.98]	0.002*
***Random Effects***	**Coef**	
Estimated Variance of the Intercept	0.26 [0.02–4.20]	0.484
**ICC**	0.072	

*OR* Odds ratio, *CI* Confidence interval, *Coef*. Coefficient, *POC-cHbA1c* Point-of-care capillary glycated hemoglobin, *ICC* Intra-class correlation

^*^p < 0.05

**p < 0.001

Table 1 presents the results of a two-level mixed-effects logistic regression model for the uptake rate of HbA1c testing, adjusting for patient characteristics and accounting for clustering at the clinic level. An unconditional means model was run to assess the need for multilevel modelling. Substantial evidence of clustering at the clinic level justified the use of multilevel logistic regression

The fixed effects include the intervention condition and patient characteristics, with random intercepts for clinics to account for within-clinic correlation

Odds Ratios (OR) represent the change in odds of HbA1c testing uptake per unit change in predictor, with 95% confidence intervals (CI) provided for each OR

#### Overall detection rate

Baseline overall detection odds (OR = 0.01, 95% CI [0.00–0.02], *p* < 0.001) were significant in the main adjusted model (Table 2). POC-cHbA1c demonstrated 99% higher odds of detecting type 2 diabetes or IFG/IGT compared to vHbA1c (OR = 1.99, 95% CI [1.01–3.95], *p* = 0.048). *Age* (OR = 1.04, 95% CI [1.02–1.06], *p* < 0.001), *history of gestational diabetes* (OR = 3.67, 95% CI [1.34–10.03], *p* = 0.012), and *obesity* (OR = 2.76, 95% CI [1.68–4.54], *p* < 0.001) significantly influenced the overall detection rate.

**Table 2:** Results of the two-level mixed-effects logistic regression model for the overall detection rate of type 2 diabetes or pre-diabetes

	***Overall Detection Rate***^a^	
	**OR [95% CI]**	***p***
***Fixed Effects***		
Intercept	0.01 [0.00–0.02]	0.001**
Intervention Condition (POC-cHbA1c)	1.99 [1.01–3.95]	0.048*
Gender (Female)	1.48 [0.93–2.37]	0.100
Age	1.04 [1.02–1.06]	0.001**
First-Degree Relative with Diabetes (Yes)	1.48 [0.93–2.36]	0.100
History of Gestational Diabetes (Yes)	3.67 [1.34–10.03]	0.012*
Hypertension (Yes)	0.86 [0.53–1.41]	0.549
Impaired Fasting Glucose (Yes)	1.53 [0.61–3.87]	0.366
Impaired Glucose Tolerance (Yes)	1.71 [0.15–19.18]	0.663
Hyperlipidemia (Yes)	1.49 [0.89–2.47]	0.126
Obesity (Yes)	2.76 [1.68–4.54]	0.001**
***Random Effects***	**Coef**	
Estimated Variance of the Intercept	0.07 [0.00–3.97]	0.628
**ICC**	0.021	

*OR* odds ratio, *CI* confidence interval, *Coef.* Coefficient, *POC-cHbA1c* Point-of-care capillary glycated hemoglobin, *ICC* Intra-class correlation

^*^p < 0.05

**p < 0.001

^a^Overall Detection Rate = diagnosed with type 2 diabetes or pre-diabetes (impaired fasting glucose/impaired glucose tolerance)

Table 2 presents the results of a two-level mixed-effects logistic regression model for the overall detection rate of type 2 diabetes or pre-diabetes, adjusting for patient characteristics and accounting for clustering at the clinic level. An unconditional means model was run to assess the need for multilevel modelling. Substantial evidence of clustering at the clinic level justified the use of multilevel logistic regression

The fixed effects include the intervention condition and patient characteristics, with random intercepts for clinics to account for within-clinic correlation

Odds Ratios (OR) represent the change in odds of overall detection per unit change in predictor, with 95% confidence intervals (CI) provided for each OR

#### Confirmatory OGTT uptake rate

Likewise, baseline OGTT uptake rate (OR = 0.05, 95% CI [0.02–0.14], *p* < 0.001) was significant in the main adjusted model (Table 3). POC-cHbA1c was associated with a statistically significant 74% higher likelihood of OGTT uptake versus vHbA1c (OR = 1.74, 95% CI [1.00–3.02], *p* = 0.049). *Age* (OR = 1.02, 95% CI [1.01–1.04], *p* = 0.002), *a first-degree relative with diabetes* (OR = 1.59, 95% CI [1.13–2.24], *p* = 0.008), *hyperlipidemia* (OR = 1.73, 95% CI [1.18–2.54], *p* = 0.005), and *obesity* (OR = 2.09, 95% CI [1.42–3.07], *p* < 0.001) significantly increased the odds of OGTT uptake, while *hypertension* (OR = 0.68, 95% CI [0.47–0.98], *p* = 0.037) significantly decreased the odds.

**Table 3:** Results of the two-level mixed-effects logistic regression model for the uptake rate of OGTT

	***OGTT Uptake Rate***	
	**OR [95% CI]**	***p***
***Fixed Effects***		
Intercept	0.05 [0.02–0.14]	0.001**
Intervention Condition (POC-cHbA1c)	1.74 [1.00–3.02]	0.049*
Gender (Female)	1.15 [0.83–1.59]	0.406
Age	1.02 [1.01–1.04]	0.002*
First-Degree Relative with Diabetes (Yes)	1.59 [1.13–2.24]	0.008*
History of Gestational Diabetes (Yes)	1.34 [0.53–3.39]	0.540
Hypertension (Yes)	0.68 [0.47–0.98]	0.037*
Impaired Fasting Glucose (Yes)	0.94 [0.42–2.08]	0.874
Impaired Glucose Tolerance (Yes)	0.69 [0.06–7.46]	0.762
Hyperlipidemia (Yes)	1.73 [1.18–2.54]	0.005*
Obesity (Yes)	2.09 [1.42–3.07]	0.001**
***Random Effects***	**Coef**	
Estimated Variance of the Intercept	0.05 [0.00–1.46]	0.555
**ICC**	0.016	

*OR*Odds ratio, *CI* Confidence interval, *Coef.* Coefficient, *OGTT* Oral glucose tolerance test, *POC-cHbA1c* Point-of-care capillary glycated hemoglobin, *ICC* Intra-class correlation

^*^p < 0.05

**p < 0.001

Table 3 presents the results of a two-level mixed-effects logistic regression model for the uptake rate of OGTT, adjusting for patient characteristics and accounting for clustering at the clinic level. An unconditional means model was run to assess the need for multilevel modelling. Substantial evidence of clustering at the clinic level justified the use of multilevel logistic regression

The fixed effects include the intervention condition and patient characteristics, with random intercepts for clinics to account for within-clinic correlation

Odds Ratios (OR) represent the change in odds of OGTT uptake per unit change in predictor, with 95% confidence intervals (CI) provided for each OR

#### Subgroup analyses

Subgroup analyses were performed to assess differences in HbA1c screening uptake based on gender and age group using Chi-squared tests. The results indicated that female participants were significantly more likely to undergo HbA1c testing compared to male participants (χ^2^(1, *N* = 852) = 4.929, *p* = 0.026). However, the interpretation of this finding should consider the unequal distribution of participants by gender (532 females; 320 males). In contrast, no significant differences in screening uptake were observed across age groups. Additional demographic information was only collected following participants' provision of informed consent, therefore the differences in HbA1c uptake among other demographic subgroups were not analyzed.

Subgroup analyses assessed differences in overall detection rate (diagnosed with type 2 diabetes or pre-diabetes) between gender and age groups using Chi-squared tests. However, no significant differences were found. Finally, subgroup analyses were performed to assess differences in confirmatory OGTT uptake based on gender and age group using Chi-squared tests. While there was no significant gender difference, participants who were aged 56–75 years were significantly more likely to undergo confirmatory OGTT testing (χ^2^ (5, *N* = 852) = 14.292, *p* = 0.014). However, a greater proportion of participants were aged 56–75 years, therefore the interpretation of this finding should take this into consideration.

## Discussion

Our study is the first to our knowledge to evaluate the real-world effectiveness of a 2-step active opportunistic screening strategy using POC-cHbA1c testing compared to vHbA1c in enhancing type 2 diabetes detection among at-risk public primary care patients in HK. Consistent with our study hypotheses, the intervention group demonstrated a greater proportion of type 2 diabetes detection, stemming from high uptake rates of POC-cHbA1c testing and subsequent confirmatory OGTT.

### Factors associated with high uptake rate

The uptake rate of POC-cHbA1c testing in the intervention group was notably high at 76.0%, surpassing that of vHbA1c. This underscores the potential of POC-cHbA1c testing to bolster screening participation, thereby facilitating early type 2 diabetes detection and intervention. Our analysis revealed a substantial 7.06 higher odds of uptake associated with POC-cHbA1c testing versus vHbA1c, supporting its acceptability and feasibility. This corresponds with prior literature indicating community-based POC-cHbA1c as a well-accepted screening test for patients [19, 20], highlighting its time-efficient and feasible nature [18]. The higher uptake of POC-cHbA1c testing can also be attributed to its logistical advantages and its alignment with the capability and opportunity domains of the “Capability, Opportunity, Motivation and Behavior” (COM-B) model for behavior change [36, 37]. Unlike vHbA1c, which requires separate appointments and a waiting period for results, POC-cHbA1c provides immediate results during the same clinic visit. This convenience enhances both the physical and psychological capability of patients to participate in screening by reducing the effort and complexity involved. Simultaneously, it increases the opportunity for participation by streamlining the process and eliminating barriers, such as scheduling conflicts or logistical delays. These factors significantly enhance the likelihood of screening uptake, while also reducing the perceived burden of testing and improving patient satisfaction [22], consistent with findings from previous studies [37, 38].

Current logistical constraints in HK GOPCs, such as the need for separate appointments for vHbA1c testing due to staffing and scheduling limitations, may introduce biases in the comparison. Moreover, even in the event of the implementation of same-day laboratory testing, delays in obtaining vHbA1c results would still remain a challenge. These contextual factors should be considered when interpreting the observed discrepancies in uptake rates. Nonetheless, the substantially higher uptake of POC-cHbA1c testing highlights its attractiveness and potential to streamline the screening process, particularly in primary care settings facing logistical challenges like those in HK.

Our study’s secondary outcomes provided additional insights into screening efficiency and patient characteristics influencing uptake rates. The NNS was 61, signifying that for every 61 individuals screened with POC-cHbA1c, one additional type 2 diabetes case was identified compared to vHbA1c. Due to a dearth of information on the NNS to detect one additional type 2 diabetes case using other diabetes screening methods, the clinical significance of our relatively small NNS becomes evident when contextualized against the NNS results of other minimally invasive screening modalities. For instance, among adult general outpatients in a medium tuberculosis (TB) incidence setting in Cameroon, the NNS using chest x-ray (CXR) only was 121 to detect one additional case of active TB [35, 39]. Whereas in a medium TB incidence setting in China, the NNS using CXR only was 1,653 among diabetic patients being screened for TB [35, 40]. Our study’s lower NNS underscores the efficiency of our screening approach, highlighting its potential value in clinical practice. A future research direction would be to determine the cost-effectiveness of this screening strategy for type 2 diabetes compared to other approaches currently used in local practice (e.g., FPG). Furthermore, the NNS should be interpreted alongside other factors. Our logistic regression analyses thus delved into factors influencing screening uptake.

*Hyperlipidemia*, *obesity* or having *a first-degree relative with diabetes* emerged as significant type 2 diabetes risk factors increasing patients’ willingness to undergo HbA1c testing and subsequent OGTT as indicated. Individuals with a family history of diabetes may be more motivated to undergo early screening through awareness of their susceptibility [41]. Likewise, hyperlipidemia and obesity are common co-morbidities [24, 28] often coexisting with insulin resistance [42], which could drive heightened awareness and motivation. This implies that individuals with a higher predisposition to type 2 diabetes are more inclined to pursue confirmatory testing, aligning with literature on how family history and individual risk profiles shape screening behaviors [21, 41]. The finding that *hypertension* decreased the odds of patients taking up HbA1c testing and OGTT may be related to the unique Risk Factor Assessment and Management Program for Hypertension (RAMP-HT) offered by HK’s GOPCs for hypertensive patients and through which they receive annual low-cost type 2 diabetes screening using FPG [43]. Presumably, such hypertensive patients would be reluctant to undergo additional HbA1c testing and OGTT if they are already being screened regularly through RAMP-HT.

Following POC-cHbA1c testing, a noteworthy trend was identified – a higher proportion of intervention group participants opted for OGTT compared to those in control clinics. This suggests that POC-cHbA1c testing has a cascade effect, potentially increasing participants’ willingness to undergo diagnostic testing. The immediacy of receiving a result after undergoing POC-cHbA1c testing in the same clinic visit may contribute to this phenomenon by reducing the delay between screening and diagnosis, keeping participants engaged and motivated to complete the diagnostic pathway. Additionally, receiving on-the-spot feedback about elevated HbA1c levels could heighten patients’ awareness of their potential risk, prompting them to take the next step toward a definitive diagnosis. This streamlined process also removes logistical barriers and facilitates real-time communication between patients and healthcare providers, facilitating personalized advice that encourages compliance when healthcare professionals recommend confirmatory OGTT. This echoes a systematic review’s findings that two steps prior to diagnostic screening are more effective in encouraging type 2 diabetes/pre-diabetes screening behavior [25]. The DiabetRisk study also supported a combined questionnaire plus POC-HbA1c strategy for identifying those with undiagnosed type 2 diabetes/pre-diabetes [44]. *Gender* showed no significant effects on both HbA1c and OGTT uptake rates, while *age* was significant only in the OGTT uptake rate, contrary to existing literature [8, 45, 46]. While *gender* [45] and *age* [8, 46] are often considered important factors in healthcare utilization and the decision-making process, their lack of significant influence on concurrent HbA1c and OGTT uptake in this context supports the assertion that type 2 diabetes risk factors play a more prominent role. This highlights the need for more tailored, person-centered screening that considers individual risk profiles.

### Type 2 diabetes/Pre-diabetes detection

A significant difference in type 2 diabetes detection was observed in the intervention versus the control group. POC-cHbA1c detected a higher proportion of cases (18 additional cases (4.2%) of previously undiagnosed type 2 diabetes detected) compared to vHbA1c (1.4%), highlighting its effectiveness in type 2 diabetes detection. This detection rate using POC-cHbA1c among the HK at-risk primary care population was in line with prior similar studies employing POC-cHbA1c conducted elsewhere [47]. Examples include the UK (2 additional cases (4.4%) of previously undiagnosed type 2 diabetes detected) [46], Spain (11 additional cases (5.9%) of previously undiagnosed diabetes detected) [48], Australia (72 additional cases (1.5%) of previously undiagnosed type 2 diabetes detected) [49] and among the remote Australian Aboriginal population (19 additional cases (7.5%) of previously undiagnosed diabetes detected) [50]. Furthermore, approximately one-third of participants who underwent confirmatory OGTT were noted to have pre-diabetes. This suggests that POC-cHbA1c can serve as a valuable tool for type 2 diabetes screening plus detection [19–21] and has the capacity to identify pre-diabetes [17, 18]. Our results align with prior evidence showing POC-cHbA1c’s efficacy in detecting undiagnosed pre-diabetes across populations [17, 18].

### Clinical implications

Our findings underscore the potential of POC-cHbA1c to enhance current type 2 diabetes screening in primary care settings, with several clinical implications. *First*, it offers robust evidence for healthcare providers and policymakers that POC-cHbA1c is a well-accepted and feasible type 2 diabetes screening test. POC-cHbA1c may be perceived as more convenient, timely, and less intimidating compared to attending another blood taking appointment for vHbA1c [36, 37], encouraging greater patient uptake. The availability of POC-cHbA1c in GOPCs could offer greater accessibility and patient satisfaction [22], which aligns with other studies conducted in primary care settings in Western countries [37]. *Second*, providing patients with information on their type 2 diabetes risk and immediate feedback through POC-cHbA1c testing could reinforce the compliance of those at risk to undergo confirmatory OGTT and may motivate them to take proactive steps towards managing their health, especially if they have known risk factors or a family history of diabetes. *Third*, POC-cHbA1c improves type 2 diabetes detection among busy hard-to-reach populations, potentially leading to earlier management and decreasing the likelihood of micro-/macro-vascular complications, as well as reducing the psychosocial burdens on patients, healthcare services, and society. *Fourth*, its ability to help identify those with pre-diabetes can facilitate the initiation of patient education, preventive measures, and lifestyle modifications, potentially delaying or even preventing the onset of type 2 diabetes [17, 44]. However, additional research is required into the cost-effectiveness and long-term clinical outcomes of POC-cHbA1c testing, as well as to explore targeted interventions to address disparities in screening uptake among different sociodemographic groups. Furthermore, the role of POC-cHbA1c as a suitable screening and diagnostic test for type 2 diabetes warrants exploration in broader populations. Investigating its popularity and widespread adoption as a detection method represents an important avenue for future research. In summary, proactive screening using POC-cHbA1c could support earlier detection of type 2 diabetes, which may facilitate timely intervention. Nonetheless, as discussed above, further research is needed to assess its potential long term impact and cost-effectiveness.

### Strengths and limitations

The consideration of contextual factors was a strength which solidified our findings by making them more generalizable and reflective of real-world settings. Also, the randomization by clinics rather than individuals minimized contamination between intervention and control clinics, enhancing the integrity of our results. However, as a limitation, this last point could have caused selection bias in the case that the intervention and control clinics for a certain cluster existed within areas of strongly differing socioeconomic or demographic profiles. Further research could help to resolve this issue. Our study has other limitations including challenges in participant and healthcare provider blinding, which may introduce bias as the intervention’s nature became evident to both physicians and patients. Lastly, although we did exceed the minimum sample size required, the relatively small sample size of this cRCT may limit the generalizability of our findings and might have also hindered our ability to detect subgroup differences adequately.

## Conclusions

Our study offers valuable insights into the effectiveness of POC-cHbA1c testing in active opportunistic screening for type 2 diabetes among the at-risk HK primary care population. The higher uptake rates of POC-cHbA1c testing and the greater proportion of type 2 diabetes detected versus vHbA1c highlights its potential as an effective tool to enhance diabetes screening coverage, expedite identification, and facilitate early treatment.

## Supplementary Information

Additional file 1: Table S1. Selected intervention and control clinics from 4 participating clusters and ethics approval information. Table S2. Multicollinearity test tolerance and VIF for outcomes: uptake rate, overall detection rate and OGTT uptake rate. Table S3. Characteristics of eligible subjects recruited in intervention and control groups. Table S4. Characteristics of enrolled subjects in intervention and control clinics. Table S5. Characteristics of eligible patients with HbA1c ≥ 5.6% in intervention and control clinics. Table S6. Characteristics of eligible high-risk patients (HbA1c ≥ 5.6%) who underwent OGTT in intervention and control clinics. Table S7. Intra-class coefficients (ICC) calculated from individual unconditional means models. Fig. 1. Study Flow Diagram. Appendix 1. Study Inclusion and Exclusion Criteria. Appendix 2. Protocol for Point-of-care Capillary HbA1c, Venous HbA1c and Oral Glucose Tolerance Tests. Appendix 3. CONSORT Guidelines for Study.

## Data Availability

The datasets generated and/or analyzed during the current study are not publicly available due to access restrictions placed on the data but are available from the corresponding author on reasonable request.

## Abbreviations

ADA: American Diabetes Association
ARR: Absolute risk reduction
BMI: Body mass index
CDCC: Chronic Disease Co-Care
CI: Confidence intervals
cRCT: Cluster randomized controlled trial
FPG: Fasting plasma glucose
GOPCs: General out-patient clinics
HbA1c: Glycated hemoglobin
HK: Hong Kong
ICC: Intra-class correlations
IDF: International Diabetes Federation
IFG: Impaired fasting glucose
IGT: Impaired glucose tolerance
NNS: Number-needed-to-screen
OGTT: Oral glucose tolerance test
OR: Odds ratios
POC-cHbA1c: Point-of-care capillary glycated hemoglobin
RAMP-HT: Risk Factor Assessment and Management Program for Hypertension
vHbA1c: Venous HbA1c
WHO: World Health Organization
