# Supplementary material for: Improving type 2 diabetes detection among at-risk individuals – comparing the effectiveness of active opportunistic screening using spot capillary-HbA1c testing and venous HbA1c testing: a cluster randomized controlled trial
Source: BMC Med. 2025 Mar 31;23:190. doi: 10.1186/s12916-025-04007-z (PMC11959842; doi:10.1186/s12916-025-04007-z)
Supplement: Supplementary file 1 — Additional file 1: Table S1. Selected intervention and control clinics from 4 participating clusters and ethics approval information. Table S2. Multicollinearity test tolerance and VIF for outcomes: uptake rate, overall detection rate and OGTT uptake rate. Table S3. Characteristics of eligible subjects recruited in intervention and control groups. Table S4. Characteristics of enrolled subjects in intervention and control clinics. Table S5. Characteristics of eligible patients with HbA1c ≥ 5.6% in intervention and control clinics. Table S6. Characteristics of eligible high-risk patients (HbA1c ≥ 5.6%) who underwent OGTT in intervention and control clinics. Table S7. Intra-class coefficients (ICC) calculated from individual unconditional means models. Fig. 1. Study Flow Diagram. Appendix 1. Study Inclusion and Exclusion Criteria. Appendix 2. Protocol for Point-of-care Capillary HbA1c, Venous HbA1c and Oral Glucose Tolerance Tests. Appendix 3. CONSORT Guidelines for Study. [file 12916_2025_4007_MOESM1_ESM.docx]

**Table S1. Selected intervention and control clinics from 4 participating clusters and ethics approval information**

| **Clusters** | **Institutional Review Board and Ethical Approval Reference** | **Selected Clinics** | **Intervention or control** | **Recruitment Period** |
| --- | --- | --- | --- | --- |
| Hong Kong West | Institutional Review Board of the University of Hong Kong / Hospital Authority Hong Kong West Cluster (reference no. UW 21-363) | Aberdeen Jockey Club GOPC | Intervention | Jun 2022 – 22 Oct 2022 |
|  |  | Sai Ying Pun GOPC | Control | Aug 2022 – 3 May 2023 |
| Kowloon Central | Research Ethics Committee Kowloon Central / Kowloon East (reference no. KC/KE-21-0137/ER-2) | Yau Ma Tei Jockey Club GOPC | Control | Jan 2023 – 2 Jul 2023 |
|  |  | Li Po Chun GOPC | Intervention | Jan 2023 – 12 Aug 2023 |
| New Territories | Joint Chinese University of Hong Kong-New Territories East Cluster Clinical Research Ethics Committee (reference no. 2021.283) | Ma On Shan Family Medicine Centre | Control | Apr 2023 – 5 Oct 2023 |
|  |  | Lek Yuen GOPC | Intervention | Apr 2023 – 27 Oct 2023 |
| Kowloon East | Research Ethics Committee Kowloon Central / Kowloon East (reference no. KC/KE-21-0144/ER-2) | Kwun Tong Community Health Centre | Intervention | 8 Nov 2023 – 8 Dec 2023 |
|  |  | Tseung Kwan O Jockey Club GOPC | Control | 8 Nov 2023 – 12 Dec 2023 |

*Note.* GOPC=General out-patient clinic.

**Table S2. Multicollinearity test tolerance and VIF for outcomes: uptake rate, overall detection rate and OGTT uptake rate**

| **Variables** | **Tolerance** | **VIF** |
| --- | --- | --- |
| Intervention Condition | 0.96 | 1.04 |
| Clinic | 0.95 | 1.06 |
| Gender | 0.97 | 1.03 |
| Age* | 0.85 | 1.18 |
| First-Degree Relative with Diabetes* | 0.96 | 1.05 |
| History of Gestational Diabetes* | 0.96 | 1.04 |
| Hypertension* | 0.86 | 1.17 |
| Impaired Fasting Glucose* | 0.95 | 1.06 |
| Impaired Glucose Tolerance* | 0.96 | 1.05 |
| Hyperlipidemia* | 0.88 | 1.14 |
| Obesity* | 0.91 | 1.10 |

*Note.* VIF=Variance inflation factor; OGTT=Oral glucose tolerance test.

*Type-2 diabetes risk factors included in step 1 of the active opportunistic risk factor screening process.

All variables are binary variables (with the exception of Age, which is a continuous variable).

VIF values above 5.0 were excluded due to the presence of significant multicollinearity.

**Table S3. Characteristics of eligible subjects recruited in intervention and control groups**

| **Demographics (*n*, %)** | Intervention (*N* = 433) | Control (*N* = 419) | *p* |
| --- | --- | --- | --- |
| **Gender** |  |  | 0.054 |
| Male | 149 (34.4%) | 171 (40.8%) |  |
| Female | 284 (65.6%) | 248 (59.2%) |  |
| Age (mean ± SD) | 61.4 ± 12.3 | 60.2 ± 13.2 | 0.400 |
|  |  |  |  |
| **Risk factors for DM* (*n*, %)** |  |  |  |
| Age ≥ 45 years | 396 (91.5%) | 367 (87.6%) | 0.065 |
| First-degree relative with DM | 118 (27.3%) | 131 (31.3%) | 0.198 |
| History of GDM | 16 (3.7%) | 8 (1.9%) | 0.115 |
| Hypertension^†^ | 154 (35.6%) | 129 (30.8%) | 0.139 |
| Obesity^†^ | 95 (21.9%) | 79 (18.9%) | 0.264 |
| Impaired fasting glucose^†^ | 16 (3.7%) | 18 (4.3%) | 0.654 |
| Impaired glucose tolerance^†^ | 1 (0.2%) | 3 (0.7%) | 0.300 |
| Hyperlipidemia^†^ | 91 (21.0%) | 90 (21.5%) | 0.869 |
|  |  |  |  |
| **Number of DM risk factors per subject (mean ± SD)**  **Number of DM risk factors per subject (*n*, %)** | 2 ± 1 | 2 ± 1 | 0.835 |
| 1 risk factor | 161 (37.2%) | 180 (43.0%) | 0.085 |
| 2 risk factors | 141 (32.6%) | 126 (30.1%) | 0.433 |
| ≥ 3 risk factors | 131 (30.3%) | 113 (27.0%) | 0.289 |

*Note.* SD=Standard deviation; DM=Diabetes mellitus; GDM=Gestational diabetes mellitus.

*Reported by patients.

^†^Hypertension was either reported by patients or determined by on-site measurement with systolic blood pressure ≥140 mmHg and/or diastolic blood pressure ≥90 mmHg; Obesity was either reported by patients or determined by on-site measurement with body mass index (BMI) ≥25 kg/m^2^ (BMI is calculated as weight (kg)/height (m)^2^); Impaired fasting glucose was reported by patients with fasting glucose concentration between 5.6-6.9 mmol/L; Impaired glucose tolerance was reported by patients with 2-hour post-challenge plasma glucose concentration between 7.8-11.0 mmol/L or HbA1c between 5.6-6.4%; Hyperlipidemia was reported by patients with total cholesterol ≥5.2 mmol/L, triglycerides ≥1.7 mmol/L or on therapy.

*P-*values are presented to indicate the statistical significance of differences between groups. A significance level of <0.05 was used. Between-group differences for categorical variables were assessed using chi-squared tests. Continuous variables were tested for normality using the Shapiro-Wilk test and for equality of variances using Levene’s test. If assumptions of normality and equal variance were satisfied, independent samples t-tests were used for analysis. If these assumptions were not met, Mann-Whitney U tests were applied. All tests were two-tailed.

#### **Table S4. Characteristics of enrolled subjects in intervention and control clinics**

|  | **Intervention (*N*=330)** | **Control (*N*=190)** | ***p*** |
| --- | --- | --- | --- |
| **Sociodemographics (n, %)** |  |  |  |
| Gender |  |  | 0.121 |
| Male | 106 (32.1%) | 74 (38.9%) |  |
| Female | 224 (67.9%) | 116 (61.1%) |  |
| Age (mean ± SD), years | 61 ± 12 | 60 ± 11 | 0.375 |
| Ethnicity |  |  | 0.506 |
| Chinese | 323 (97.9%) | 188 (98.9%) | 0.366 |
| Indian | 2 (0.6%) | – | 0.282 |
| Filipino | 5 (1.5%) | 2 (1.1%) | 0.657 |
| Education level |  |  | **<0.001** |
| No-schooling/Pre-primary | 9 (2.7%) | – | **0.021** |
| Primary Level | 76 (23.0%) | 25 (13.2%) | **0.006** |
| Secondary Level | 189 (57.3%) | 111 (58.4%) | 0.777 |
| Post-secondary or above | 56 (17.0%) | 54 (28.4%) | **0.002** |
| Employment status |  |  | 0.768 |
| Not working | 162 (49.1%) | 91 (47.9%) |  |
| Working | 168 (50.9%) | 99 (52.1%) |  |
| Working hours of employed subjects (mean ± SD),  hours per day | 8 ± 2.4 | 8 ± 1.9 | 0.941 |
| Household income, HKD/month |  |  | **0.049** |
| Below 5000 | 62 (18.8%) | 16 (8.4%) | **0.001** |
| 5000 - 19999 | 81 (24.5%) | 54 (28.4%) | 0.303 |
| 20000 - 39999 | 83 (25.2%) | 47 (24.7%) | 0.901 |
| 40000 or above | 40 (12.1%) | 32 (16.8%) | 0.137 |
| Do not know | 64 (19.4%) | 41 (21.6%) | 0.561 |
| Comprehensive Social Security Assistance | |  | 0.909 |
| No | 319 (96.7%) | 184 (96.8%) |  |
| Yes | 11 (3.3%) | 6 (3.2%) |  |
|  |  |  |  |
| **Health service utilization, n (%)** |  |  |  |
| Have family doctor |  |  | 0.880 |
| No | 266 (80.6%) | 152 (80.0%) |  |
| Yes | 64 (19.4%) | 38 (20.0%) |  |
| History of check-up |  |  | 0.480 |
| No | 194 (58.8%) | 106 (55.8%) |  |
| Yes | 136 (41.2%) | 84 (44.2%) |  |
| History of blood glucose testing |  |  | 0.212 |
| No | 208 (63.0%) | 109 (57.4%) |  |
| Yes | 122 (37.0%) | 81 (42.6%) |  |
|  |  |  |  |
| **Smoking status, n (%)** |  |  | 0.832 |
| Non-smoker | 279 (84.5%) | 164 (86.3%) | 0.575 |
| Ex-smoker | 23 (7.0%) | 11 (5.8%) | 0.594 |
| Current smoker | 28 (8.5%) | 15 (7.9%) | 0.806 |
|  |  |  |  |
| **Level of physical activity*, n (%)** |  |  |  |
| Inactive | 207 (62.7%) | 108 (56.8%) | 0.172 |
| Minimally active | 92 (27.9%) | 63 (33.2%) | 0.172 |
| HEPA active | 31 (9.4%) | 19 (10.0%) | 0.741 |
|  |  |  |  |
| **Frequency of alcohol consumption, n (%)** |  |  | 0.154 |
| Never | 280 (84.8%) | 148 (77.9%) | **0.037** |
| Monthly or less | 28 (8.5%) | 18 (9.5%) | 0.621 |
| Weekly or less | 12 (3.6%) | 12 (6.3%) | 0.163 |
| ≥ 2 times per week | 10 (3.0%) | 12 (6.3%) | 0.074 |

**Table S4. Characteristics of enrolled subjects in intervention and control clinics (Cont’)**

|  | **Intervention (*N*=330)** | **Control (*N*=190)** | ***p*** |
| --- | --- | --- | --- |
| **Anthropometric measurements (mean ± SD)** |  | |  |
| SBP (mm/Hg) | 137 ± 17 | 132 ± 19 | **0.002** |
| DBP (mm/Hg) | 76 ± 12 | 77 ± 12 | 0.478 |
| Weight (kg) | 62 ± 12 | 62 ± 12 | 0.959 |
| Height (cm) | 160 ± 8 | 160 ± 9 | 0.406 |
| WC (cm) | 86 ± 11 | 85 ± 12 | 0.926 |
| HC (cm) | 97 ± 7 | 96 ± 8 | 0.246 |
|  |  |  |  |
| **BMI**^†^**, n (%)** |  |  |  |
| BMI (mean ± SD) | 24 ± 4 | 24 ± 4 | **0.036** |
| Underweight (BMI < 18.5 kg/m^2^) | 20 (6.1%) | 6 (3.2%) | 0.159 |
| Healthy weight (BMI = 18.5 – 22.9 kg/m^2^) | 103 (31.2%) | 64 (33.7%) | 0.577 |
| Overweight (BMI = 23.0 – 24.9 kg/m^2^) | 74 (22.4%) | 43 (22.6%) | 0.889 |
| Obese (BMI ≥ 25.0 kg/m^2^) | 127 (38.5%) | 63 (33.2%) | 0.215 |
|  |  |  |  |
| **WHR**^‡^**, n (%)** |  |  |  |
| WHR (mean ± SD) | 1 ± 0 | 1 ± 0 | **0.042** |
| Central obesity (Male: WHR > 0.9/WC > 85cm;   Female: WHR > 0.8/WC > 80cm) | 250 (75.8%) | 132 (69.5%) | 0.676 |
| Normal | 79 (23.9%) | 44 (23.2%) | 0.676 |
|  |  |  |  |
| **HbA1c (mean ± SD), %** | 5.6 ± 0 | 5.7 ± 0 | **<0.001** |
|  |  |  |  |
| **Confirmatory OGTT**^§^ **(mean ± SD), mmol/L** |  |  |  |
| FPG | 5.2 ± 1 | 5.2 ± 1 | 0.733 |
| 2hPG | 7.8 ± 3 | 7.0 ± 2 | **0.023** |
|  |  |  |  |

*Note.* Data is presented as mean ± SD or n (proportion).

SD=Standard deviation; SBP=Systolic blood pressure; DBP=Diastolic blood pressure; WC=Waist circumference; HC=Hip circumference; BMI=Body Mass Index; WHR=Waist-hip ratio; HbA1c=Glycated hemoglobin; OGTT=Oral glucose tolerance test; FPG= Fasting plasma glucose; 2hPG= 2-hour plasma glucose.

*The Guidelines of International Physical Activity Questionnaire (IPAQ) is adopted. Minimally inactive refers to a) 3 or more days of vigorous activity of at least 20 minutes per day OR b) 5 or more days of moderate-intensity activity or walking of at least 30 minutes per day OR c) 5 or more days of any combination of walking, moderate-intensity or vigorous intensity activities achieving a minimum of at least 600 metabolic equivalent of task (MET)-minutes/week. Health-enhancing physical activity (HEPA) refers to a) vigorous-intensity activity on at least 3 days and accumulating at least 1500 MET-minutes/week OR b) 7 or more days of any combination of walking, moderate-intensity or vigorous intensity activities achieving a minimum of at least 3000 MET-minutes/week. Inactive refers to a) no activity is reported OR b) some activity is reported but not enough to meet criteria for “minimally inactive” or “HEPA active”. MET-minutes/week is calculated as 3.3 × walking minutes × days/week + 4.0 × moderate-intensity activity minutes × moderate-intensity days/week + 8.0 × vigorous-intensity activity minutes × vigorous-intensity days/week.

^†^BMI is calculated as weight (kg)/height (m)^2^.

^‡^WHR is calculated as WC (cm)/HC (cm).

^§^Subjects with HbA1c ≥5.6% would be invited for confirmatory OGTT. 145 participants (out of 176 subjects with HbA1c ≥5.6%) from intervention clinics and 95 participants from control clinics have taken OGTT (out of 115 subjects with HbA1c ≥5.6%).

*P-*values are presented to indicate the statistical significance of differences between groups. A significance level of <0.05 was used. Between-group differences for categorical variables were assessed using chi-squared tests. Continuous variables were tested for normality using the Shapiro-Wilk test and for equality of variances using Levene’s test. If assumptions of normality and equal variance were satisfied, independent samples t-tests were used for analysis. If these assumptions were not met, Mann-Whitney U tests were applied. All tests were two-tailed.

#### **Table S5. Characteristics of eligible patients with HbA1c ≥5.6% in intervention and control clinics**

|  | | **Intervention (*N*=176)** | **Control (*N*=115)** | ***p*** |
| --- | --- | --- | --- | --- |
| **Sociodemographics (n, %)** | |  |  |  |
| Gender | |  |  | 0.599 |
| Male | | 59 (33.5%) | 42 (36.5%) |  |
| Female | | 117 (66.5%) | 73 (63.5%) |  |
| Age (mean ± SD), years | | 64 ± 11 | 62 ± 11 | 0.273 |
| Ethnicity | |  |  | 0.496 |
| Chinese | | 173 (98.3%) | 114 (99.1%) | 0.550 |
| Indian | | 2 (1.1%) | – | 0.251 |
| Filipino | | 1 (0.6%) | 1 (0.9%) | 0.761 |
| Education level | |  |  | **0.002** |
| No-schooling/Pre-primary | | 3 (1.7%) | ­– | 0.159 |
| Primary Level | | 46 (26.1%) | 14 (12.2%) | **0.004** |
| Secondary Level | | 103 (58.5%) | 70 (60.9%) | 0.690 |
| Post-secondary or above | | 24 (13.6%) | 31 (27.0%) | **0.005** |
| Employment status | |  |  | 0.898 |
| Not working | | 92 (52.3%) | 61 (53.0%) |  |
| Working | | 84 (47.7%) | 54 (47.0%) |  |
| Working hours of employed subjects (mean ± SD), hours | | 8 ± 2.5 | 8 ± 2.0 | 0.630 |
| Household income, HKD/month | |  |  | 0.525 |
| Below 5000 | | 32 (18.2%) | 11 (9.6%) | **0.043** |
| 5000 - 19999 | | 41 (23.3%) | 33 (28.7%) | 0.301 |
| 20000 - 39999 | | 41 (23.3%) | 31 (27.0%) | 0.479 |
| 40000 or above | | 23 (13.1%) | 16 (13.9%) | 0.836 |
| Do not know | | 39 (22.2%) | 24 (20.9%) | 0.794 |
| Comprehensive Social Security Assistance |  | | | 0.394 |
| No | | 170 (96.6%) | 113 (98.3%) |  |
| Yes | | 6 (3.4%) | 2 (1.7%) |  |
|  | |  |  |  |
| **Health service utilization, n (%)** | |  |  |  |
| Have family doctor | |  |  | 0.209 |
| No | | 138 (78.4%) | 97 (84.3%) |  |
| Yes | | 38 (21.6%) | 18 (15.7%) |  |
| History of check-up | |  |  | 0.729 |
| No | | 97 (55.1%) | 61 (53.0%) |  |
| Yes | | 79 (44.9%) | 54 (47.0%) |  |
| History of blood glucose testing | |  |  | 0.357 |
| No | | 106 (60.2%) | 63 (54.8%) |  |
| Yes | | 70 (39.8%) | 52 (45.2%) |  |
|  | |  |  |  |
| **Smoking status, n (%)** | |  |  | 0.484 |
| Non-smoker | | 149 (84.7%) | 103 (89.6%) | 0.230 |
| Ex-smoker | | 13 (7.4%) | 6 (5.2%) | 0.464 |
| Current smoker | | 14 (8.0%) | 6 (5.2%) | 0.367 |
|  | |  |  |  |
| **Level of physical activity*, n (%)** | |  |  |  |
| Inactive | | 116 (65.9%) | 63 (54.8%) | 0.057 |
| Minimally active | | 48 (27.3%) | 38 (33.0%) | 0.057 |
| HEPA active | | 12 (6.8%) | 14 (12.2%) | 0.117 |
|  | |  |  |  |
| **Frequency of alcohol consumption, n (%)** | |  |  | 0.466 |
| Never | | 148 (84.1%) | 97 (84.3%) | 0.953 |
| Monthly or less | | 17 (9.7%) | 6 (5.2%) | 0.170 |
| Weekly or less | | 6 (3.4%) | 6 (5.2%) | 0.448 |
| ≥ 2 times per week | | 5 (2.8%) | 6 (5.2%) | 0.299 |

**Table S5. Characteristics of eligible patients with HbA1c ≥5.6% in intervention and control clinics (Cont’)**

|  | **Intervention (*N*=176)** | **Control (*N*=115)** | ***p*** |
| --- | --- | --- | --- |
| **Anthropometric measurements (mean ± SD)** |  | |  |
| SBP (mm/Hg) | 138 ± 17 | 133 ± 18 | **0.007** |
| DBP (mm/Hg) | 76 ± 12 | 77 ± 11 | 0.617 |
| Weight (kg) | 64 ± 13 | 62 ± 13 | 0.218 |
| Height (cm) | 159 ± 9 | 160 ± 9 | 0.559 |
| WC (cm) | 88 ± 11 | 86 ± 12 | 0.357 |
| HC (cm) | 99 ± 7 | 96 ± 9 | **0.016** |
|  |  |  |  |
| **BMI**^†^**, n (%)** |  |  |  |
| BMI (mean ± SD) | 25 ± 4 | 24 ± 4 | **0.046** |
| Underweight (BMI < 18.5 kg/m^2^) | 11 (6.3%) | 3 (2.6%) | 0.156 |
| Healthy weight (BMI = 18.5 – 22.9 kg/m^2^) | 42 (23.9%) | 39 (33.9%) | 0.061 |
| Overweight (BMI = 23.0 – 24.9 kg/m^2^) | 41 (23.3%) | 26 (22.6%) | 0.976 |
| Obese (BMI ≥ 25.0 kg/m^2^) | 79 (44.9%) | 45 (39.1%) | 0.332 |
|  |  |  |  |
| **WHR**^‡^**, n (%)** |  |  |  |
| WHR (mean ± SD) | 1 ± 0 | 1 ± 0 | 0.352 |
| Central obesity (Male: WHR > 0.9/WC > 85cm;   Female: WHR > 0.8/WC > 80cm) | 143 (81.3%) | 92 (80.0%) | 0.791 |
| Normal | 33 (18.8%) | 23 (20.0%) | 0.791 |
|  |  |  |  |
| **HbA1c (mean ± SD), %** | 5.9 ± 0 | 5.9 ± 0 | 0.560 |
|  |  |  |  |
| **Confirmatory OGTT**^§^ **(mean ± SD), mmol/L** |  |  |  |
| FPG | 5.2 ± 1 | 5.2 ± 1 | 0.733 |
| 2hPG | 7.8 ± 3 | 7.0 ± 2 | **0.023** |
|  |  |  |  |

*Note.* Data is presented as mean ± SD or n (proportion).

SD=Standard deviation; SBP=Systolic blood pressure; DBP=Diastolic blood pressure; WC=Waist circumference; HC=Hip circumference; BMI=Body Mass Index; WHR=Waist-hip ratio; HbA1c=Glycated hemoglobin; OGTT=Oral glucose tolerance test; FPG= Fasting plasma glucose; 2hPG= 2-hour plasma glucose.

*The Guidelines of International Physical Activity Questionnaire (IPAQ) is adopted. Minimally inactive refers to a) 3 or more days of vigorous activity of at least 20 minutes per day OR b) 5 or more days of moderate-intensity activity or walking of at least 30 minutes per day OR c) 5 or more days of any combination of walking, moderate-intensity or vigorous intensity activities achieving a minimum of at least 600 metabolic equivalent of task (MET)-minutes/week. Health-enhancing physical activity (HEPA) refers to a) vigorous-intensity activity on at least 3 days and accumulating at least 1500 MET-minutes/week OR b) 7 or more days of any combination of walking, moderate-intensity or vigorous intensity activities achieving a minimum of at least 3000 MET-minutes/week. Inactive refers to a) no activity is reported OR b) some activity is reported but not enough to meet criteria for “minimally inactive” or “HEPA active”. MET-minutes/week is calculated as 3.3 × walking minutes × days/week + 4.0 × moderate-intensity activity minutes × moderate-intensity days/week + 8.0 × vigorous-intensity activity minutes × vigorous-intensity days/week.

^†^BMI is calculated as weight (kg)/height (m)^2^.

^‡^WHR is calculated as WC (cm)/HC (cm).

^§^Subjects with HbA1c ≥5.6% would be invited for confirmatory OGTT. 145 participants (out of 176 subjects with HbA1c ≥5.6%) from intervention clinics and 95 participants from control clinics have taken OGTT (out of 115 subjects with HbA1c ≥5.6%).

*P-*values are presented to indicate the statistical significance of differences between groups. A significance level of <0.05 was used. Between-group differences for categorical variables were assessed using chi-squared tests. Continuous variables were tested for normality using the Shapiro-Wilk test and for equality of variances using Levene’s test. If assumptions of normality and equal variance were satisfied, independent samples t-tests were used for analysis. If these assumptions were not met, Mann-Whitney U tests were applied. All tests were two-tailed.

#### **Table S6. Characteristics of eligible high-risk patients (HbA1c ≥5.6%) who underwent OGTT in intervention and control clinics**

|  | | **Intervention (*N*=145)** | **Control (*N*=95)** | ***p*** |
| --- | --- | --- | --- | --- |
| **Sociodemographics (n, %)** | |  |  |  |
| Gender | |  |  | 0.447 |
| Male | | 48 (33.1%) | 36 (37.9%) |  |
| Female | | 97 (66.9%) | 59 (62.1%) |  |
| Age (mean ± SD), years | | 63 ± 11 | 62 ± 11 | 0.770 |
| Ethnicity | |  |  | 0.688 |
| Chinese | | 143 (98.6%) | 94 (98.9%) | 0.824 |
| Indian | | 1 (0.7%) | – | 0.417 |
| Filipino | | 1 (0.7%) | 1 (1.1%) | 0.762 |
| Education level | |  |  | **0.029** |
| No-schooling/Pre-primary | | 1 (0.7%) | – | 0.417 |
| Primary Level | | 39 (26.9%) | 13 (13.7%) | **0.013** |
| Secondary Level | | 82 (56.6%) | 56 (58.9%) | 0.714 |
| Post-secondary or above | | 23 (15.9%) | 26 (27.4%) | **0.031** |
| Employment status | |  |  | 0.729 |
| Not working | | 73 (50.3%) | 50 (52.6%) |  |
| Working | | 72 (49.7%) | 45 (47.4%) |  |
| Working hours of employed subjects (mean ± SD), hours | | 8 ± 2.4 | 8 ± 1.9 | 0.715 |
| Household income, HKD/month | |  |  | 0.838 |
| Below 5000 | | 26 (17.9%) | 10 (10.5%) | 0.116 |
| 5000 - 19999 | | 33 (22.8%) | 27 (28.4%) | 0.322 |
| 20000 - 39999 | | 33 (22.8%) | 24 (25.3%) | 0.656 |
| 40000 or above | | 22 (15.2%) | 14 (14.7%) | 0.926 |
| Do not know | | 31 (21.4%) | 20 (21.1%) | 0.952 |
| Comprehensive Social Security Assistance |  | | | 0.548 |
| No | | 142 (97.9%) | 94 (98.9%) |  |
| Yes | | 3 (2.1%) | 1 (1.1%) |  |
|  | |  |  |  |
| **Health service utilization, n (%)** | |  |  |  |
| Have family doctor | |  |  | **0.036** |
| No | | 109 (75.2%) | 82 (86.3%) |  |
| Yes | | 36 (24.8%) | 13 (13.7%) |  |
| History of check-up | |  |  | 0.585 |
| No | | 80 (55.2%) | 49 (51.6%) |  |
| Yes | | 65 (44.8%) | 46 (48.4%) |  |
| History of blood glucose testing | |  |  | 0.743 |
| No | | 84 (57.9%) | 53 (55.8%) |  |
| Yes | | 61 (42.1%) | 42 (44.2%) |  |
|  | |  |  |  |
| **Smoking status, n (%)** | |  |  | 0.370 |
| Non-smoker | | 123 (84.8%) | 86 (90.5%) | 0.198 |
| Ex-smoker | | 12 (8.3%) | 6 (6.3%) | 0.573 |
| Current smoker | | 10 (6.9%) | 3 (3.2%) | 0.211 |
|  | |  |  |  |
| **Level of physical activity*, n (%)** | |  |  |  |
| Inactive | | 93 (64.1%) | 50 (52.6%) | 0.076 |
| Minimally active | | 42 (29.0%) | 33 (34.7%) | 0.076 |
| HEPA active | | 10 (6.9%) | 12 (12.6%) | 0.132 |
|  | |  |  |  |
| **Frequency of alcohol consumption, n (%)** | |  |  | 0.086 |
| Never | | 122 (84.1%) | 78 (82.1%) | 0.679 |
| Monthly or less | | 17 (11.7%) | 5 (5.3%) | 0.090 |
| Weekly or less | | 3 (2.1%) | 6 (6.3%) | 0.090 |
| ≥ 2 times per week | | 3 (2.1%) | 6 (6.3%) | 0.090 |

**Table S6. Characteristics of eligible high-risk patients (HbA1c ≥5.6%) who underwent OGTT in intervention and control clinics (Cont’)**

|  | **Intervention (*N*=145)** | **Control (*N*=95)** | ***p*** |
| --- | --- | --- | --- |
| **Anthropometric measurements (mean ± SD)** |  | |  |
| SBP (mm/Hg) | 137 ± 16 | 134 ± 18 | 0.157 |
| DBP (mm/Hg) | 76 ± 12 | 78 ± 11 | 0.337 |
| Weight (kg) | 64 ± 12 | 63 ± 13 | 0.381 |
| Height (cm) | 159 ± 8 | 160 ± 9 | 0.353 |
| WC (cm) | 88 ± 11 | 87 ± 12 | 0.497 |
| HC (cm) | 99 ± 7 | 96 ± 9 | 0.055 |
|  |  |  |  |
| **BMI**^†^**, n (%)** |  |  |  |
| BMI (mean ± SD) | 25 ± 4 | 24 ± 4 | 0.080 |
| Underweight (BMI < 18.5 kg/m^2^) | 7 (4.8%) | – | **0.030** |
| Healthy weight (BMI = 18.5 – 22.9 kg/m^2^) | 36 (24.8%) | 34 (35.8%) | 0.068 |
| Overweight (BMI = 23.0 – 24.9 kg/m^2^) | 32 (22.1%) | 21 (22.1%) | 0.793 |
| Obese (BMI ≥ 25.0 kg/m^2^) | 67 (46.2%) | 39 (41.1%) | 0.432 |
|  |  |  |  |
| **WHR**^‡^**, n (%)** |  |  |  |
| WHR (mean ± SD) | 1 ± 0 | 1 ± 0 | 0.316 |
| Central obesity (Male: WHR > 0.9/WC > 85cm;   Female: WHR > 0.8/WC > 80cm) | 117 (80.7%) | 76 (80.0%) | 0.895 |
| Normal | 28 (19.3%) | 19 (20.0%) | 0.895 |
|  |  |  |  |
| **HbA1c (mean ± SD), %** | 5.9 ± 0 | 5.9 ± 0 | 0.663 |
|  |  |  |  |
| **Confirmatory OGTT**^§^ **(mean ± SD), mmol/L** |  |  |  |
| FPG | 5.2 ± 1 | 5.2 ± 1 | 0.733 |
| 2hPG | 7.8 ± 3 | 7.0 ± 2 | **0.023** |
|  |  |  |  |

*Note.* Data is presented as mean ± SD or n (proportion).

SD=Standard deviation; SBP=Systolic blood pressure; DBP=Diastolic blood pressure; WC=Waist circumference; HC=Hip circumference; BMI=Body Mass Index; WHR=Waist-hip ratio; HbA1c=Glycated hemoglobin; OGTT=Oral glucose tolerance test; FPG= Fasting plasma glucose; 2hPG= 2-hour plasma glucose.

*The Guidelines of International Physical Activity Questionnaire (IPAQ) is adopted. Minimally inactive refers to a) 3 or more days of vigorous activity of at least 20 minutes per day OR b) 5 or more days of moderate-intensity activity or walking of at least 30 minutes per day OR c) 5 or more days of any combination of walking, moderate-intensity or vigorous intensity activities achieving a minimum of at least 600 metabolic equivalent of task (MET)-minutes/week. Health-enhancing physical activity (HEPA) refers to a) vigorous-intensity activity on at least 3 days and accumulating at least 1500 MET-minutes/week OR b) 7 or more days of any combination of walking, moderate-intensity or vigorous intensity activities achieving a minimum of at least 3000 MET-minutes/week. Inactive refers to a) no activity is reported OR b) some activity is reported but not enough to meet criteria for “minimally inactive” or “HEPA active”. MET-minutes/week is calculated as 3.3 × walking minutes × days/week + 4.0 × moderate-intensity activity minutes × moderate-intensity days/week + 8.0 × vigorous-intensity activity minutes × vigorous-intensity days/week.

^†^BMI is calculated as weight (kg)/height (m)^2^.

^‡^WHR is calculated as WC (cm)/HC (cm).

^§^Subjects with HbA1c ≥5.6% would be invited for confirmatory OGTT. 145 participants (out of 176 subjects with HbA1c ≥5.6%) from intervention clinics and 95 participants from control clinics have taken OGTT (out of 115 subjects with HbA1c ≥5.6%).

*P-*values are presented to indicate the statistical significance of differences between groups. A significance level of <0.05 was used. Between-group differences for categorical variables were assessed using chi-squared tests. Continuous variables were tested for normality using the Shapiro-Wilk test and for equality of variances using Levene’s test. If assumptions of normality and equal variance were satisfied, independent samples t-tests were used for analysis. If these assumptions were not met, Mann-Whitney U tests were applied. All tests were two-tailed.

**Table S7. Intra-class coefficients (ICC) calculated from individual unconditional means models**

| **Target Variable** | **ICC** |
| --- | --- |
| HbA1c Uptake Rate | 0.311 |
| Overall Detection Rate (Type 2 diabetes/Pre-diabetes) | 0.067 |
| Oral Glucose Tolerance Test Uptake Rate | 0.047 |

*Note*. ICC values were calculated from initial individual unconditional means models for each target variable.

ICC values above 0.05 indicate substantial clustering effects.

**Fig. 1. Study Flow Diagram**

**Recruitment**

**Primary care patients**

To be recruited from 8 different public primary care clinics in Hong Kong

To exclude if meet any exclusion criteria

**776 Eligible patients who provided written consent to join study**

**Intervention clinics**

**(N=388; from 4 clinics, 97/clinic)**

- *On-site* POC-cHbA1c testing offered
- Immediate face-to-face notification of HbA1c level and risk of DM

**If HbA1c ≥5.6%**

- On-site immediate invitation to schedule OGTT visit at the same clinic within 2–4 weeks.

**Control clinics**

**(N=388; from 4 clinics, 97/clinic)**

- vHbA1c testing offered, to be scheduled on a *separate clinic visit* within 2 weeks
- Inform of HbA1c level and DM risk by phone within a week of results being available

**If HbA1c ≥5.6%**

- Phone invitation to schedule OGTT visit at the same clinic within 2–4 weeks.

**Step 2 HbA1c screen for abnormal glycemic status**

**(Random allocation by clinic; 1:1)**

**Follow-up OGTT**

🡪 **DM** if FG ≥7.0mmol/L +/or 2hPG ≥11.1mmol/L

**Outcome measures**

1^o^ Difference in proportion of T2DM detected between intervention and control groups

1^o^ Uptake rate of POC-cHbA1c testing and vHbA1c testing among consented participants

2^o^ Number-needed-to-screen for POC-cHbA1c to detect one more case with T2DM compared to vHbA1c testing

2^o^ Proportion of high HbA1c ≥5.6% among the studied at-risk group

2^o^ Difference in proportion of OGTT diagnostic test uptake between groups

**Follow-up OGTT**

🡪 **DM** if FG ≥7.0mmol/L +/or 2hPG ≥11.1mmol/L

**Analyses**

**Outcome measures**

**Step 3 Confirmatory** **OGTT for DM**

**if HbA1c ≥5.6%**

**Enrollment**

**Eligibility screen**

**Inclusion criteria (any of the below):**

**- Age ≥45 years**

**- First-degree relatives with DM**

**- History of Gestational DM**

**- Hypertension**

**- IFG/IGT**

**- Hyperlipidemia,** **or**

**- Obesity (BMI ≥25 kg/m^2^)**

**Step 1**

**Active Opportunistic Risk Factor Screen**

Exclusion criteria:

- Known DM / on OHA Rx
- Received DM screening test within

12 months
- Pregnant / breast-feeding
- Active thyroid diseases or anemia
- On iron / systemic steroid Rx
- History of blood donation / blood
 transfusion within 3 months

*Note*. T2DM=Type 2 diabetes; DM=Diabetes mellitus; IFG=Impaired fasting glucose; IGT=Impaired glucose tolerance; BMI=Body mass index; OHA=Oral hypoglycemic agent; Rx=Medical prescription; POC-cHbA1c=Point-of-care capillary glycated hemoglobin; vHbA1c=Venous glycated hemoglobin; OGTT=Oral glucose tolerance test; FPG=Fasting plasma glucose; 2hPG=2-hour plasma glucose.

**Appendix 1. Study Inclusion and Exclusion Criteria**

***Inclusion Criteria (any of the following):***

1. Age ≥45 years;
2. Family history of diabetes mellitus (DM) in a first-degree relative;
3. History of Gestational DM;
4. Hypertension (i.e. Systolic Blood Pressure (BP) ≥140 mmHg, Diastolic BP ≥90 mmHg or on therapy for hypertension);
5. History of pre-diabetes (i.e. impaired fasting glucose with fasting glucose concentration between 5.6-6.9 mmol/L, impaired glucose tolerance with 2-hour post challenge plasma glucose concentration between 7.8-11.0 mmol/L or HbA1c between 5.6-6.4%);
6. Hyperlipidemia (i.e. total cholesterol ≥5.2 mmol/L, triglycerides ≥1.7 mmol/L or on therapy);
7. Obesity (i.e. body mass index (BMI) ≥25 kg/m^2^).

***Exclusion Criteria (any of the following):***

1. Known history of type 2 diabetes or on hypoglycemic treatment;
2. Received type 2 diabetes screening within 12-months.
3. Women who are pregnant or breast-feeding at recruitment;
4. Active thyroid diseases (including subjects on thyroid replacement therapy or anti-thyroid drugs) or active endocrine diseases such as Cushing's syndrome or Acromegaly at recruitment;
5. History of blood donation or blood transfusion within 3 months prior to recruitment;
6. Patients who are taking systemic steroid therapy or iron supplement at recruitment.

**Appendix 2. Protocol for Point-of-care Capillary HbA1c, Venous HbA1c and Oral Glucose Tolerance Tests**

**1. Hemoglobin A1c (HbA1c) Test**

Hemoglobin A1c, also known as glycated hemoglobin, reflected the average blood sugar levels over the past three months. It could be measured by venous or capillary methods.

**2. Point-of-care Capillary HbA1c (POC-cHbA1c) Test**

The POC-cHbA1c machine employed in this study was the Cobas b 101 system (Roche Diagnostics, Switzerland). Testing was carried out by nurses and researchers who had been trained by the Cobas b 101 machine supplier in the use of the POC-cHbA1c machine and internal quality assurance processes. This training encompassed proper device operation, sample collection techniques, adherence to quality control protocols, and troubleshooting procedures. Participants were advised to wash their hands with non-alcoholic soap and to dry them properly. A capillary blood sample was obtained by dermal puncture of a fingertip using a disposable lancet and resorbed in the specially marked area of the Cobas b 101 test disc. The disc was then inserted into the autoanalyzer. Test results displayed in both percentage and mmol/L were available within 10 minutes.

To ensure validity and reliability of the POC-cHbA1c test result, measurements were performed with the NGSP-certified Cobas b 101 machine that measured HbA1c values by immunoassays, and internal plus external quality assurance tests of the machine were conducted regularly according to the protocol described below.

***2.1. Internal Quality Assurance (to be performed at every session)***

Before each session, an optical test and quality control test were conducted for internal quality assurance. The name of the nurse/researcher performing the test, the test date and result were recorded for internal reference.

2.1.1. Optical Test

The optical test was designed to check the optical function and accuracy of the entire machine. If the test was passed successfully, the optical functions of the machine were considered to be working normally.

Depending on the machine, an optical check disc (or optical test cartridge) was provided. The control test was run by inserting the disc (or cartridge) into the compartment until the test was completed. It then displayed whether the test was a pass or fail (or a value was displayed that needed to be checked whether it fell within the normal range as indicated). Only when a pass (or normal value) was obtained in the optical test could the nurse/researcher proceed to the Quality Control test.

2.1.2. Quality Control (QC) Test

The QC test ensured the operation technique and results obtained from the machine were accurate. The QC solutions had defined values with the acceptable ranges listed on the HbA1c QC info disc (or QC value card), which was unique in each QC lot.

The following materials were required for the test:

- an HbA1c QC info disc (or QC value card);

- two QC reagents with normal and abnormal HbA1c levels;

- HbA1c test disc (or capillary holder and cartridge).

*2.1.2.1. Procedures for QC Test:*

1. The QC information on target values and ranges were imported by inserting the QC info disc (or QC value card) into the machine.
2. One of the QC reagents was applied to the suction point of the HbA1c test disc (or capillary holder).
3. After making sure it was completely filled, any excess control solution was removed carefully with a lint-free tissue.
4. The disc (or capillary holder inserted in a cartridge) was placed into the compartment and the test was run.
5. When it was completed, steps 2 to 4 were repeated using the other QC reagents.
6. When both tests were finished, the discs (or cartridges) were discarded appropriately.

Only when the results from both tests fell within acceptable ranges would the nurse or researcher proceed with using the Cobas b 101 machine for testing patients.

***2.2. External Quality Assurance (every month)***

2.2.1. Proficiency Test

The proficiency test (PT) served to verify if the operation technique, reagents, system, and testing performance of the Cobas b 101 machine were comparable with external operators. A supporting laboratory provided samples whose values were unknown to the trained nurse or researcher performing the test and the results from different nurses or researchers were analyzed against each other. A monthly report with peer comparisons using the same machine and method was generated for users’ reference.

The test was performed once per month. Each of the involved nurses or researchers performed the PT at least once throughout the project.

*2.2.1.1. Procedures for PT:*

1. Samples were delivered by the external operator according to a schedule and were stored unopened at 2-8°C. They were labelled with the date by which the results were to be submitted.
2. According to the labelled date, tests were conducted according to usual practice for true assessment of the operation performance.
3. The name of the nurse or researcher performing the test, the test date and result were recorded for internal reference.
4. The results were submitted electronically via an online platform.

The monthly report with peer comparisons together with the internal records were used for review. If needed, further training was required for those with unsatisfactory performance.

**3. Venous HbA1c (vHbA1c) Test**

Non-fasting venous blood samples were collected by trained nurses for the measurement of vHbA1c concentrations. The samples were sent to a laboratory accredited by the Hong Kong Accreditation Service and/or an international college of pathologists (e.g., The College of American Pathologists or The Royal College of Pathologists of Australasia). High-performance liquid chromatography was used for measurement of vHbA1c concentrations. The results were available within 7 days.

**4. Oral Glucose Tolerance Test (OGTT)**

This test measured the body’s ability to absorb glucose after consumption of a specific amount of sugar. It was demonstrated by the increase in blood sugar levels from fasting to 2 hours after ingestion of a 75-gram glucose solution.

Before the scheduled test, participants were reminded to:

1. Continue a normal diet for 2-3 days before the blood test.
2. Fast for at least 8 hours before the scheduled test. They could drink water but were advised to avoid other beverages including coffee and caffeinated tea.

During the test, participants first underwent venipuncture to test for the fasting glucose level. Upon completion, the participants were then given a 300 ml glucose solution containing 75 grams of glucose. The glucose load was to be consumed within 5 minutes and the participants were instructed to remain seated and to refrain from smoking plus eating until the second venous blood sampling occurred after 2 hours. The paired blood samples were sent to a laboratory accredited by the Hong Kong Accreditation Service and/or an international college of pathologists (e.g., The College of American Pathologists or The Royal College of Pathologists of Australasia). Plasma glucose concentrations were measured by the Hexokinase method to determine the glycemic status of the participants.

**Appendix 3. CONSORT Guidelines for Study**

| Section/Topic | Item No | Checklist item | Reported on page No |
| --- | --- | --- | --- |
| Title and abstract | | | |
|  | 1a | Identification as a randomized trial in the title | 1 |
|  | 1b | Structured summary of trial design, methods, results, and conclusions (for specific guidance see CONSORT for abstracts) | 3-4 |
| Introduction | | | |
| Background and objectives | 2a | Scientific background and explanation of rationale | 5-7 |
|  | 2b | Specific objectives or hypotheses | 6-7 |
| Methods | | | |
| Trial design | 3a | Description of trial design (such as parallel, factorial) including allocation ratio | 7-8 |
|  | 3b | Important changes to methods after trial commencement (such as eligibility criteria), with reasons | N/A |
| Participants | 4a | Eligibility criteria for participants | 7, Additional file 1: Appendix 1 |
|  | 4b | Settings and locations where the data were collected | 7-8, Additional file 1: Table S1 |
| Interventions | 5 | The interventions for each group with sufficient details to allow replication, including how and when they were actually administered | 10-12, Additional file 1: Appendix 2 |
| Outcomes | 6a | Completely defined pre-specified primary and secondary outcome measures, including how and when they were assessed | 13 |
|  | 6b | Any changes to trial outcomes after the trial commenced, with reasons | N/A |
| Sample size | 7a | How sample size was determined | 8-10 |
|  | 7b | When applicable, explanation of any interim analyses and stopping guidelines | N/A |
| Randomization: |  |  |  |
| Sequence generation | 8a | Method used to generate the random allocation sequence | 7-8 |
|  | 8b | Type of randomization; details of any restriction (such as blocking and block size) | 7-8 |
| Allocation concealment mechanism | 9 | Mechanism used to implement the random allocation sequence (such as sequentially numbered containers), describing any steps taken to conceal the sequence until interventions were assigned | 7-8 |
| Implementation | 10 | Who generated the random allocation sequence, who enrolled participants, and who assigned participants to interventions | 7 |
| Blinding | 11a | If done, who was blinded after assignment to interventions (for example, participants, care providers, those assessing outcomes) and how | N/A |
|  | 11b | If relevant, description of the similarity of interventions | N/A |
| Statistical methods | 12a | Statistical methods used to compare groups for primary and secondary outcomes | 13-15 |
|  | 12b | Methods for additional analyses, such as subgroup analyses and adjusted analyses | 13-15 |
| Results | | | |
| Participant flow (a diagram is strongly recommended) | 13a | For each group, the numbers of participants who were randomly assigned, received intended treatment, and were analyzed for the primary outcome | Fig. 1 |
|  | 13b | For each group, losses and exclusions after randomization, together with reasons | Fig. 1 |
| Recruitment | 14a | Dates defining the periods of recruitment and follow-up | Additional file 1: Table S1 |
|  | 14b | Why the trial ended or was stopped | N/A |
| Baseline data | 15 | A table showing baseline demographic and clinical characteristics for each group | Additional file 1: Table S3 |
| Numbers analyzed | 16 | For each group, number of participants (denominator) included in each analysis and whether the analysis was by original assigned groups | Fig. 1 |
| Outcomes and estimation | 17a | For each primary and secondary outcome, results for each group, and the estimated effect size and its precision (such as 95% confidence interval) | 15-19 |
|  | 17b | For binary outcomes, presentation of both absolute and relative effect sizes is recommended | N/A |
| Ancillary analyses | 18 | Results of any other analyses performed, including subgroup analyses and adjusted analyses, distinguishing pre-specified from exploratory | 16-20 |
| Harms | 19 | All important harms or unintended effects in each group (for specific guidance see CONSORT for harms) | N/A |
| Discussion | | | |
| Limitations | 20 | Trial limitations, addressing sources of potential bias, imprecision, and, if relevant, multiplicity of analyses | 26 |
| Generalizability | 21 | Generalizability (external validity, applicability) of the trial findings | 26 |
| Interpretation | 22 | Interpretation consistent with results, balancing benefits and harms, and considering other relevant evidence | 20-26 |
| Other information | | |  |
| Registration | 23 | Registration number and name of trial registry | 4, 15 |
| Protocol | 24 | Where the full trial protocol can be accessed, if available | ClinicalTrials.gov (NCT06382363) and The University of Hong Kong Clinical Trials registries (HKUCTR-3002) |
| Funding | 25 | Sources of funding and other support (such as supply of drugs), role of funders | 28 |
